# Supplementary material for: Development of a Targeted NGS Assay for the Detection of Respiratory Pathogens including SARS-CoV-2 in Felines
Source: Pathogens. 2024 Apr 17;13(4):335. doi: 10.3390/pathogens13040335 (PMC11055025; doi:10.3390/pathogens13040335)
Supplement: Supplementary file 1 [file pathogens-13-00335-s001.zip › Supplementary tables S1, S2, S3.pdf]

Table S1. Diagnostic Sensitivity and Specificity Testing- comparison between qPCR and tNGS assay for detection of feline respiratory pathogens from 31 clinical samples.

**ACCESSION**	**SPECIMEN**	**qPCR Results**			**tNGS results**
A13-4105	lung	FeHV-1 Ct	16.43	Other pathogens not tested	Results matched qPCR results
A14-7200	lung	FeHV-1 Ct	20.88	Other pathogens not tested	Results matched qPCR results
S12-1183	lung	FCV Ct	19.76	Other pathogens not tested	Results matched qPCR results
A18-14725	lung	FCV Ct	16.28	Other pathogens not tested	Results matched qPCR results
A19-8904	Nasal swab	FeHV-1 Ct	15.66	Other pathogens not tested	Results matched qPCR results
A12-603	lung	FeHV-1 Ct	17.36	Other pathogens not tested	Results matched qPCR results
A23-11429	Nasal Swab	*Mycoplasma* spp. Ct	28.33	Negative for *Chlamydia* spp., FeHV-1	**Negative for *B. bronchiseptica***, *Chlamydia felis*, FeHV-1, Positive for *Mycoplasma felis*, FCV
		B. bronchiseptica Ct	33.21		
		FCV Ct	27.54		
A23-13268	OP Swab	*Mycoplasma* spp. Ct	35.38	Negative *for B. bronchiseptica*, *Chlamydia* spp., FCV, FeHV-1	Results matched qPCR results
A24-11806	Lung	*Mycoplasma* spp. Ct	20.02	Negative for *Chlamydia* spp., FCV, FeHV-1	Results matched qPCR results
		B. bronchiseptica Ct	29.62		
A24-14404	Endotracheal Wash	*Mycoplasma* spp. Ct	33.17	Other pathogens not tested	Result matched qPCR result
A24-14405	Nasal Swab	*Mycoplasma* spp. Ct	20.33	Negative *for B. bronchiseptica*, *Chlamydia* spp., FCV, FeHV-1	Results matched qPCR results
A24-5450	Lung	*Mycoplasma* spp. Ct	31.42	Negative for *B. bronchiseptica*	**Negative for *Chlamydia felis***, *B. bronchiseptica,* Positive for *Mycoplasma felis*, FCV, and FeHV-1
		Chlamydia spp. Ct	31.33		
		FCV Ct	35.58		
		FeHV-1 Ct	29.29		
A24-5450	Nasal Swab	*Mycoplasma* spp. Ct	20.54	Negative for *B. bronchiseptica*	Results matched qPCR results
		Chlamydia spp. Ct	23.44		
		FCV Ct	29.27		
		FeHV-1 Ct	14.91		

A24-6974	Ocular, OP, and Nasal Swabs	*Mycoplasma* spp. Ct	23.56	Negative for *Chlamydia* spp.	Results matched qPCR results
		B. bronchiseptica Ct	26.78		
		FCV Ct	25.44		
		FeHV-1 Ct	35.00		
A24-6974	OP and Ocular Swabs		*	Negative for *B. bronchiseptica*, *Chlamydia* spp., FCV, FeHV-1, *Mycoplasma* spp.	Results matched qPCR results
A24-7717	OP Swab	*Mycoplasma* spp. Ct	22.76	Negative for *B. bronchiseptica*, FeHV-1	**Positive for *B. bronchiseptica***, *Mycoplasma felis*, FCV, **Negative for *Chlamydia felis*,** FeHV-1
		Chlamydia spp. Ct	30.42		
		FCV Ct	30.26		
A24-8065	Endotracheal Wash	*Mycoplasma* spp. Ct	24.89	Other pathogens not tested	Results matched qPCR result
A24-8396	OP Swab	*Mycoplasma* spp. Ct	23.19	Negative for *B. bronchiseptica*, FCV, FeHV-1	Positive for *Mycoplasma felis*, **Negative for *Chlamydia* felis**, *B. bronchiseptica*, FCV, FeHV-1
		Chlamydia spp. Ct	27.69		
A23-13578	OP Swab	*B. bronchiseptica* Ct	34.16	Negative for *Mycoplasma* spp., *Chlamydia* spp., FCV, FeHV-1	Results matched qPCR results
A24-10580	Nasal Swab	*B. bronchiseptica* Ct	33.05	Negative for *Mycoplasma* spp.	Results matched qPCR results
		Chlamydia spp. Ct	31.64		
		FCV Ct	22.20		
		FeHV-1 Ct	16.02		
A24-5813	Lung pool	*B. bronchiseptica* Ct	32.02	Negative for *Chlamydia* spp., FeHV-1	Results matched qPCR results
		Mycoplasma spp. Ct	20.23		
		FCV Ct	19.08		
A24-8280	OP Swab	*B. bronchiseptica* Ct	35.00	Negative for *Chlamydia* spp., FCV, FeHV-1, *Mycoplasma* spp.	**Negative for *B. bronchiseptica***, *Chlamydia* spp., FCV, FeHV-1**, Positive for *Mycoplasma felis***
A23-17385	OP Swab		*	Negative for *B. bronchiseptica*,	Results matched qPCR results

				Chlamydia spp., FCV, FeHV-1, *Mycoplasma* spp.	
A24-2384	Bronchoalveolar Lavage (BAL)		*	Negative for *Mycoplasma* spp.	Results matched qPCR results
A24-5509	Lung	FeHV-1 Ct	36.40	Negative for *B. bronchiseptica*, *Chlamydia* spp., FCV, *Mycoplasma* spp.	Results matched qPCR results
A24-5650	OP Swab	*Chlamydia* spp. Ct	31.97	Negative for *B. bronchiseptica* and *Mycoplasma* spp.	**Negative for *Chlamydia felis*, FCV,** positive for FeHV-1, negative *for B. bronchiseptica* and *Mycoplasma felis*
		FCV Ct	25.57		
		FeHV-1 Ct	22.16		
A24-6304	OP Swab		*	Negative for *B. bronchiseptica*, *Chlamydia* spp., FCV, FeHV-1, *Mycoplasma* spp.	Results matched qPCR results
A24-6455	Lung		*	Negative for *B. bronchiseptica*, *Chlamydia* spp., FCV, FeHV-1, *Mycoplasma* spp.	**Positive for *Mycoplasma felis***, Negative for *B. bronchiseptica, Chlamydia* spp., FCV, FeHV-1
A24-7609	Nasal Swab		*	Negative for *B. bronchiseptica*, *Chlamydia* spp., FCV, FeHV-1, *Mycoplasma* spp.	Results matched qPCR results
Cheerio	Ocular swab	*Chlamydia* spp. Ct	36.9	Other pathogens not tested	Results matched qPCR
Atticus	Ocular Swab	*Mycoplasma* spp. Ct	Positive, Ct unknown	Negative for FCV, FeHV-1	Results matched qPCR

Table S2. Comparison of MagMAX Viral/Pathogen Nucleic Acid Isolation Kit and MagMAX CORE Nucleic Acid Purification Kit with silica bead beating

**Sample 1**	Viral/Pathogen Kit (avg number reads)	CORE (avg number reads)
SARS-CoV-2	**3498**	**158**
FeHV-1	152626	61152
FCV	1054	93
C. felis	259766	113676
B. bronchiseptica	141793	67118
**Sample 2**		
SARS-CoV-2	**688**	**261**
FeHV-1	55911	63182

FCV	222	94
C. felis	86056	112225
B. bronchiseptica	46264	77783
**Sample 3**		
SARS-CoV-2	**655**	**211**
FeHV-1	56761	56829
FCV	171	53
C. felis	79931	102064
B. bronchiseptica	46319	70982
**Sample 4**		
SARS-CoV-2	**1404**	**238**
FeHV-1	118188	55572
FCV	404	35
C. felis	162247	72399
B. bronchiseptica	46264	78088

Table S3. Comparison of MagMAX Viral/Pathogen Nucleic Acid Isolation Kit vs MagMAX Pathogen RNA/DNA Kit for extraction of SARS-CoV-2 from feline positive and negative clinical samples.

Sample- Ct value by qPCR	Viral/Pathogen Kit (avg number reads)	Pathogen RNA/DNA Kit (avg number reads)
1- 35.95, 38.49 (considered inconclusive by qPCR)	30	0
2- 31.08, 30.23	4123	3650
3- 33.04, 36.49	59	243
4- No detection	0	0
5- 32.02, 31.75	757	483
6- No detection	0	0
